# Recent Advances and Future Prospects on the Tailing Covering Technology for Oxidation Prevention of Sulfide Tailings

**DOI:** 10.3390/toxics11010011

**Published:** 2022-12-22

**Authors:** Meiyan Si, Yunjian Chen, Chen Li, Yichao Lin, Jianhong Huang, Feng Zhu, Senlin Tian, Qun Zhao

**Affiliations:** 1 Faculty of Environmental Science and Engineering, Kunming University of Science and Technology, Kunming 650504, China; 2 Yunnan Geological Engineering Second Survey Institute Co., Ltd., Kunming 650213, China; 3 Faculty of Metallurgy and Environment, Central South University, Changsha 410083, China

**Keywords:** acid mine drainage, tailing covering technology, sulfide tailings, oxidation prevention

## Abstract

Acid mine drainage, produced from sulfur-containing mine waste exposed to air, water, and bacteria, is considered as a serious environmental pollutant because of its extremely low pH and excessive heavy metals. In order to solve the ecological environment problems caused by the acid mine drainage, treatment methods such as neutralization, adsorption, passivation, bio-inhibition, and physical coverage have been developed. Nevertheless, these methods are terminal treatment methods, which are unable to prevent the generation of acid mine drainage at the source. Recently, it is noteworthy that the tailing covering technology is particularly emphasized, owing to its superior source control capability. By reducing the contact with air, water, and bacteria, the oxidation of sulfide tailings is significantly reduced, thus avoiding the production of acid mine drainage. To date, massive research has been studied and parts of technologies have been applied, but the review on the principles, processes, and applications of these technologies are still lacking. Thus, the present review aims to increase the knowledge related to the most relevant application of tailing covering technology with the following aspects: (i) the background, concepts, and performance of tailing covering technology; (ii) the applicable conditions for each tailings coverage system and their advantages and limitations; and (iii) the future perspective of this technology.

## 1. Introduction

**Figure 1:**
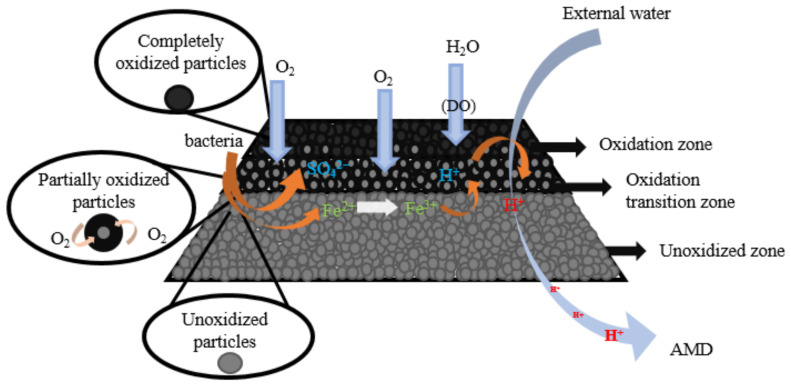
Oxidation of sulfide minerals and formation of AMD.

Continuous exploitation of mineral resources plays a great role in industrial development, but also produces massive mine solid waste. As reported, the annual discharge of mine tailings in the world has exceeded 10 billion tons [1]. The accumulation of such a large amount of tailings is a great hidden danger to the surrounding environment. Acid mine drainage (AMD), produced by the oxidation of sulfide tailings (Figure 1) [1], is considered to be the most challenging pollutant to treat because of its extremely low pH, high heavy metals content, and high sulfate concentration. Furthermore, AMD contains large quantities of toxic substances, such as cyanides, hazardous heavy metals (e.g., Cu, Zn, Cd, Mn, Pb, Cr, Ni, and Fe), and toxic metalloid (e.g., As and Se) [2,3]. Once the AMD leaks, it can pose a long-term and large-scale threat to the environment around the mining area, especially the surface water, groundwater, and soil, thereby affecting the health of the residents and the biodiversity of ecosystems [3,4,5,6,7,8,9,10]. Therefore, preventing and controlling AMD pollution are two of the top priorities of today’s global mining industry.

To solve the problem of AMD pollution, massive technologies have been proposed and applied, such as neutralization [11], sulfide precipitation [12], electrocoagulation [13], wetlands construction [14], and microbial treatment [15]. Although these methods have exhibited great performance in AMD treatment, they are all limited to taking effect after AMD has been generated and spread. Inevitably, ecological risks exist in the runoff, collection, and treatment of AMD [5]. Instead, source control to avoid the generation of AMD is a better approach, and tailing covering technology has emerged [16]. Based on the principle that the AMD is produced by the interaction of sulfur-containing tailings with water, oxygen and microbial components, various technical branches have been proposed for applicability to different mining environments and natural conditions. Through the unremitting efforts of research, so far, tailing covering technology has proved to have great advantages in AMD source control. However, the review of these technologies is still lacking. Herein, we believe that a comprehensive review of tailing covering technology and the evaluation of single technology could be key steps towards the exploration of new materials for AMD covering technology in the future.

In this review, we systematically reviewed a large number of foreign studies and studied how the covering system was implemented and worked and mapped out the previous studies and current trends of tailings covering technologies. The background, concepts, and performance of tailing covering technology are comprehensively discussed. The applicable conditions for each tailings coverage system and their advantages and limitations are systematically reviewed and compared. Furthermore, the future perspective outlook for advances in this technology is proposed.

## 2. Tailings Cover Technology

Water and oxygen are the main reactants to produce AMD, and the inhibition methods should consider controlling the availability of one or two components. Therefore, two strategies are adopted: (i) preventing oxygen from entering the tailings pile and thus reducing the rate of sulfide oxidation; and (ii) isolating the infiltration of external water and thus weakening the role of dissolved oxygen. According to different coverage principles, tailing cover systems can be divided into dry covers, wet covers, and organic covers, which are expatiated in the following sections.

### 2.1. Wet Covers

Wet covers forms a fully saturated water cover to prevent tailing oxidation caused by the advection transport and diffusion of oxygen [17], thus reducing the rate of tailing oxidation.

#### 2.1.1. Flooded Cover

**Figure 2:**
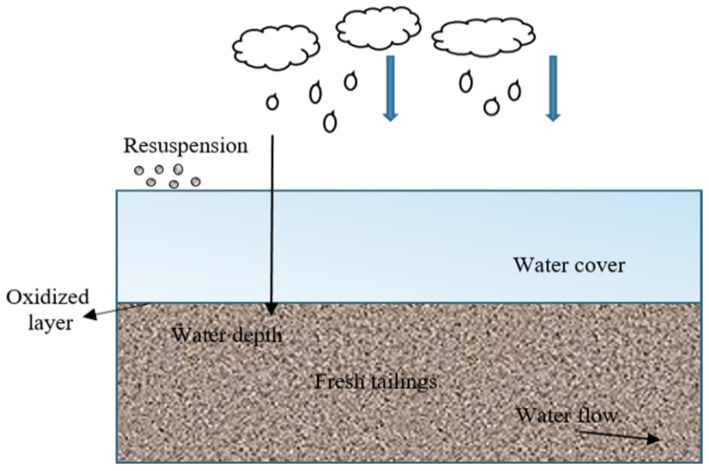
Schematic diagram of flooded cover system [22]. (Note: the thick arrow represents rainfall; the long arrow indicates the depth of water cover; the arrow on the left indicates the extension of the oxidized layer; the arrow on right indicates the runoff direction of water in the fresh tailing.) This picture was adapted from [22] with permission from publisher Elsevier in May 2016.

When sulfur-containing tailings are placed below the water surface, the low solubility (25 °C, 8.6 mg·L^−1^) and low effective diffusion coefficient (D_e_ = 2 × 10^−9^ m^2^·s^−1^) of oxygen in water can be utilized to create a natural anoxic environment, which can theoretically permanently inhibit the oxidation activity of tailings (Figure 2). Garcilaso et al. reported that the flooded cover is an efficient treatment scheme [18]. The natural isolation of water made the flux of O_2_ through the water layer to the tailings reduce by 10,000 times, and D_e_ decreases from 3.86 × 10^−6^ to 8 × 10^−12^ m^2^·s^−1^ [19,20]. Romano et al. [21] used a couples oxygen diffusion and sulfide-mineral oxidation (PYROX) model to simulate the oxidation of sulfide minerals over a 100-year period, with sulfate as the target product. Results showed that the oxidation rate of flooded cover system decreased by about 99.1% compared with open tailings.

Unfortunately, the immersed sulfide tailings are not completely inert, and the oxidation reaction continued to occur at the tailing–water interface slowly. An investigation on a historical tailing pond (Savage River mine, located in northwest Tasmania, Australia) found that there was an oxidation zone of 0.05 m under the water surface of 1.5 m, and the acidic pH values were measured of the tailings pore water, and the heavy metals increased (Ni: 76–123 mg·kg^−1^, Zn: 45–54 mg·kg^−1^, Cu: 91–679 mg·kg^−1^) [22]. Under the action of wind and waves of shallow water overburden layer, turbulence and tailing re-suspension occurred at the air–water interface, which are important reasons for the oxidation of underwater tailings [23]. In addition, the increase in interstitial water acidity (pH, from 9.5 to 8.5) and SO_4_^2−^ production just under the interface also proves that the tailings gradually oxidized [24]. Research shows that the presence of a transitional oxidation front at approximately 0.3–0.6 m across the sub-aerial zone, with interlayer oxidation tailings containing pyrite enriched in Cu, Co and Zn, is observed. Fortunately, the low sulfur alteration intensity (SAI < 2/10) makes the combined organic and water cover effectively limiting, and the risk for AMD production was low [22] To tackle this problem, Mustafa et al. [25] weakened the influence of wind by optimizing the overlying depth, thus controlling the sediment re-suspension within an acceptable range.

Flooded cover is effective as an oxygen barrier, but it needs high economic expenditure, such as water supplement, dam construction, and maintenance. More importantly, it is not suitable for arid and semi-arid areas where annual evaporation is greater than precipitation, and water supplement will greatly increase the maintenance cost in the later stage [26]. In addition, the stability of dam is a consideration, especially in earthquake-prone areas.

#### 2.1.2. Cover with Capillary Barrier Effects (CCBE)

**Figure 3:**
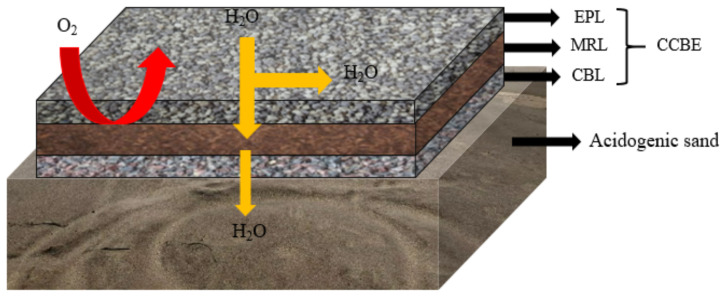
Schematic diagram of cover with capillary barrier effects.

CCBE is a typical multilayer cover mode, which consists of at least three layers of cover [27] (Figure 3). A capillary break layer (CBL) made of coarse-grained material is located at the bottom and serves as a support. The middle part is a moisture-retaining layer (MRL) made of fine particles, which has low saturated hydraulic conductivity (Ksat > 10^−5^ cm·s^−1^) and high water retention capacity to limit oxygen migration [28]. The surface layer is an evaporation protective layer (EPL) made of coarse particles, which plays a role in limiting evaporation and promoting horizontal drainage. When two kinds of particle layers with different particle sizes contact, the vertical flow between the two layers is often limited due to the difference in unsaturated hydraulic properties, resulting in a capillary barrier effect [29,30], which maintains high saturation in the layer with finer particle sizes (SR > 85%). Therefore, the substitution of various cheap materials (industrial waste, mining waste, natural materials, etc.) has become the research hotspot of CCBE.

A large number of findings have confirmed the effectiveness of CCBE in limiting oxygen diffusion (about 99%) and AMD production [31,32,33]. Dagenais et al. [34] monitored the CCBE performance data for four consecutive years and found that the MRL remained saturated for many years, with oxygen flux as low as 10 g·m^−2^·a^−1^. Larochelle et al. [35] used acid-producing waste rock as CBL to conduct laboratory column experiments. They found that the saturation of MRL remained around 85–90%, and the pH remained close to neutral during the whole test period. A similar result was obtained by Molson et al. [33], wherein the O_2_ flux was only related to the porosity of the material when the MRL layer was close to saturation, and its thickness had relatively little influence. For example, in five pilot-scale field tests (four sites were constructed with CCBE over the tailings, a site was uncovered) of the Norebec-Manitou mine site, Canada, the finite volume model MIN3P was used to simulate the reduction of AMD discharge by using the cover with CCBE [33]. The monitored data after 1200 days showed that the pH remained neutral (about 6.5~7), the concentration of SO_4_^2−^ and Fe^2+^ decreased to 1700 mg·L^−1^ and 8 mg·L^−1^, respectively, and Cu^2+^ concentrations decreased to 0.001 mg·L^−1^. Overall, 1–7 orders of magnitude are reduced compared with the uncovered.

Generally, CCBE can effectively inhibit the generation of AMD. However, in the area where evaporation is greater than precipitation, it is easy to cause excessive desaturation of MRL layer and gradually lose the function as oxygen barrier. In addition, the CCBE has a disadvantage on long-term stability. According to the report of Pabst et al. [30], the inhibition efficiency of sulfur-containing tailings decreased significantly after being covered for 10 years. More importantly, CCBE is not being an economic option. The covering materials required for its construction are usually shipped from other locations because on-site alternatives such as non-acid-producing tailings are not readily available, which greatly increases construction costs [36]. Therefore, CCBE is not the optimal coverage option in terms of efficiency and economy.

#### 2.1.3. Elevated Water Table (EWT)

**Figure 4:**
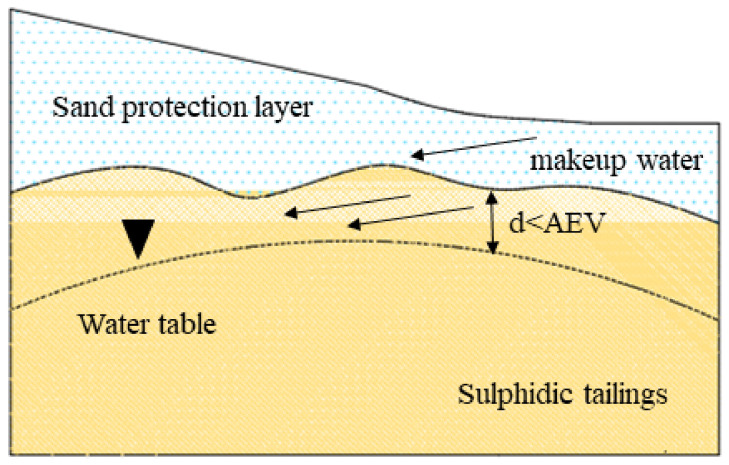
Schematic diagram of elevated water table coverage. (Note: the black triangle indicates the water level depth; the black dotted line indicates that the water level depth is less than the minimum value of AEV; and the black arrow indicates the flow direction of makeup water.

The EWT method mainly relies on raising the groundwater level to keep the depth at the edge height of the capillary effect layer to increase the saturation of the tailings [37,38,39]. Hence, controlling the height of the water level is the key to the effectiveness of this technology, and it is also the main difference from flooded cover (Figure 4). In saturated or nearly saturated porous media, the effective diffusion coefficient of oxygen is very low, resulting in a lower oxygen flux that reduces the oxidation rate of sulfide minerals [40]. Therefore, this technology is usually coupled with a monolayer cover method. Bussière et al. [41] combined EWT with monolayer cover technique to significantly inhibit sulfide mineral oxidation. Related studies have confirmed this conclusion. For example, the combination of coarse-grained covering materials and EWT could increase penetration and limit water loss caused by evaporation [40,42]. The combination with fine-grained covering materials could promote moisture retention and prevent evapotranspirative demand, and no significant tailing oxidation is observed (<10^−4^ kg·(m^2^·a^−1^)^−1^) [43,44]. Some studies have shown that there is a correlation between the depth of groundwater level and the rate of oxygen consumption [45]. Ouangrawa et al. [37] demonstrated that oxygen diffusion and oxidation of sulfide minerals can be prevented by keeping the water table depth less than the air entry value (AVE) of tailings and keeping the tailings highly saturated (Sw ≥ 90%). In the simulated profiles experiment of SO_4_^2−^ and Fe(III), within column 1, peak SO_4_^2−^ and Fe(III) concentrations reached about 12,000 mg·L^−1^ and 2000 mg·L^−1^, respectively. However, as the EWT increased, within column 6, SO_4_^2−^ and Fe(III) concentrations were significantly lower, at 3300 mg·L^−1^ and 10 mg·L^−1^, respectively. Furthermore, the observed discharge pH became more neutral (pH~7–8) [38]. The decrease in SO_4_^2−^ and Fe(III) concentrations were limited by gypsum and ferrihydrite precipitation. Similar to flooded cover and CCBE technologies, EWT technique adopts the low effective diffusion coefficient of O_2_ in saturated and near-saturated media [19,40]. The transmission of O_2_ is the main factor limiting the oxidation of sulfide minerals.

EWT technology is an effective auxiliary governance scheme, but its environmental and technical requirements are too high. The maintenance of high water level requires long-term funds investment, technical support, and personnel maintenance. The application of EWT technology can only be limited in humid areas with low evaporation, and artificial reconstruction will greatly increase the economic cost. This is a common problem with all wet cover techniques.

### 2.2. Dry Covers

Dry covers are low permeability preservative layers made of inorganic mineral materials. It can prevent oxygen and water from entering the tailings layer and reduce the oxidation rate of sulfide minerals [46]. The dry cover layer is composed of materials with different granulation characteristics, such as compacted clay, portland cement, fly ash, etc. [47].

#### 2.2.1. Monolayer Cover

**Figure 5:**
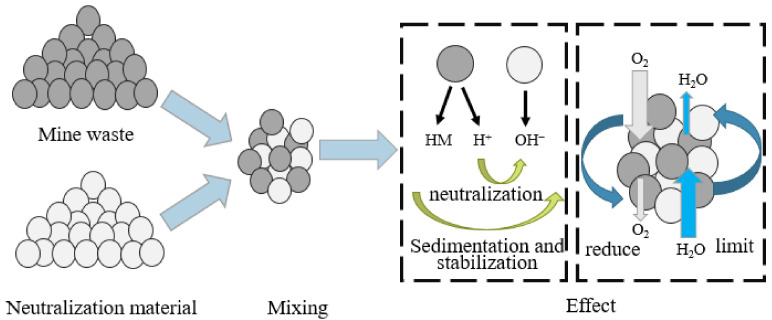
Mixing of mine waste and neutralizing materials [54]. This picture was adapted from [54] with permission from publisher Elsevier in March 2019.

Monolayer cover is the simplest and most widely used method of tailing pond cover technology. It reduces the diffusion flux of oxygen and the accumulation of acid caused by oxidation by virtue of the low porosity and chemical properties of the surface covering material, such as limestone or phosphate minerals. In practical applications, monolayer cover usually refers to a mixture of two or more materials with different properties (Figure 5). In some studies, desulfurized tailings with non-acid producing activity were used as a single material covering layer, and the oxidation rate was reduced by 75~82% [21]. However, in the face of the huge storage of sulfide minerals, the production of AMD is still huge in sufficient oxidation time. Hence, the oxygen barrier performance of single layer covering is poor. Pabst et al. [48] put forward a similar view that a single material covering layer could not prevent the oxidation of sulfide minerals in the underlying tailings. TA tailings covered with CA produce a very acidic leachate (pH < 3) and, after 10 cycles, the pH was below 2.5. Meanwhile, the concentration of sulfate and iron reaches 40,000 mg·L^−1^ and 8000 mg·L^−1^, respectively. These condition inhibitions on AMD were limited. Therefore, more research tends to use a mixture of multiple materials. For example, the blending of tailings with waste rock [49], limestone [50], and neutralizing sludge [51] all showed better barrier performance as single-layer covering materials. Hakkou et al. [52] covered the surface of acid-producing tailings with 15% of alkaline phosphate waste mixed with coarse-size tailings and found that the acidity accumulation peaked at 3199 mg CaCO_3_·L^−1^ and stabilized at 280 mg CaCO_3_·L^−1^, and sulfate concentration caused by oxidation decreased significantly, from 4900 mg·L^−1^ to 480 mg·L^−1^, in a short time.

The availability of single-layer covering materials and the simplicity of technology implementation are favored by many researchers and mine managers. However, the performance of monolayer cover mainly depends on the thickness of the cover and the properties of mixed materials. Therefore, the increase in coverage thickness also means an increase in cost. In addition, the long-term stability of monolayer cover is also a concern. If the neutralization effect of surface alkalinity on the oxidation products of the lower layer is lost, the barrier effect will be greatly weakened. At the same time, the drying of the surface material can easily lead to the cracking of the covering layer. Dehydration cracks can provide preferential channels for air and water, making them lose their barrier function, providing a preferential channel for air and moisture [53]. Therefore, monolayer cover is more of a temporary protection measure.

#### 2.2.2. Stabilization Cover

**Figure 6:**
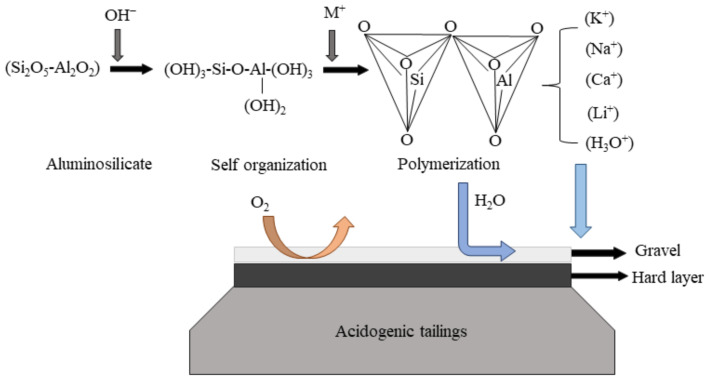
Schematic diagram of geopolymer reaction [64]. This picture was adapted from [64] with permission from publisher Elsevier in 2021.

Stabilization treatment is mainly used to solidify sulfide minerals under the action of binders. The hard layer formed by solidifying can inhibit the reactivity of sulfide and the migration of harmful components, thereby eliminating the negative impacts on the receiving environment [55]. A commonly used binder is Portland cement. However, cement cannot be directly applied to high sulfur tailings because residual acid and sulfate salts inhibit its chemical stability [56]. In addition, cement is vulnerable to harmful effects caused by sulfate attack [57,58]. Therefore, as binders for stabilization cover processes, geopolymers have been recommended as a substitute [59,60]. Under the action of an alkali activator, solid silicoaluminate dissolves and releases Si and Al monomer and dimer. Then, polymerization occurs to form a hardened material with three-dimensional structure (Figure 6). Silicoaluminate is the main component of ore, so the reuse of tailings and waste rock has become an advantage [61].

Ahn et al. [56] used the polymer prepared by lime, tailings, and sodium silicate as the covering layer of sulfur-containing tailings. The TCLP results indicate that the leached amounts of heavy metals Cd, Fe, Mn, and Zn of GP tailings were significantly reduced by 99.3%, 92.9, 98.9, and 99.7%, respectively, with 10% S/S materials compared to the original tailings. The stability of heavy metals was attributed to the carbonate-bound phases, and sulfide minerals were surrounded by calcium silicate generated from sodium silicate, inhibiting further reaction. Ash fly is rich in SiO_2_ and Al_2_O_3_, which have the potential to prepare geopolymers. The research confirmed that the geopolymers prepared from fly ash have a great advantage in stabilizing Cr(NO_3_)_3_ [62]. In addition, the hard layer tends to be stable for a long time. Sarkkinen et al. [63] used the analytic hierarchy process, including life cycle assessment, to evaluate the three stable treatment schemes (Table 1). The results showed the advantages of stabilized cover technology in economy, technology, and ecology and highlighted the characteristics of sustainability.

Stabilization cover is a relatively new method, which can physically weaken the water penetration and oxygen diffusion while solidifying and sequestration pollutants in a three-dimensional structure, thus improving the long-term availability of the covering layer. However, due to the complexity of the site environment of mine wasteland, such as runoff drainage with strong acidity and high sulfate concentration, the stability of cover should be seriously concerning, especially sulfate erosion and drying cracking.

**Table 1:** Multiple-criteria decision analysis of solidification/stabilization cover scheme [65]. This picture was adapted from [65] with permission from publisher Elsevier in 10 May 2017.

	Structure		Main Criteria Weight	Cost Accounting (€/m^2^)	Normalized Cluster Value	Priority Order
	Above the Surface	Below Surface				
SPC ^a^	Vegetation (100 mm)	PC stabilized tailings (1 m)	Economic, 33% Technical, 33% Social-Ecological, 33%	26.32	0.172	3
AAC ^b^	Vegetation (100 mm)	AAC stabilized tailings (1 m)	Economic, 33% Technical, 33% Social-Ecological, 33%	26.78	0.238	2
AHCL ^c^	Vegetation (100 mm) + Moraine (300 mm) + tailings (200 mm) + CaO + moraine (200 mm)	AAC stabilized tailings (300 mm)	Economic, 33% Technical, 33% Social-Ecological, 33%	22.09	0.262	1
Soil layer				500		
Multilayer			2000–3000			

^a^ stabilization with cement; ^b^ MgO activated ground granulated blast furnace slag; ^c^ advanced hardpan cover liner.

#### 2.2.3. Benign Material Cover

Two advantages make benign materials a competitive choice for cover layers. The first is the continuous alkaline release capacity, which reduces soluble metals and non-metals by consuming H^+^ to produce precipitation. The second is low permeability, which reduces the oxidation rate by physically reducing the oxygen flux. In recent years, industrial by-products and residues (such as green liquor slag, fly ash, cement kiln dust, red mud bauxite, etc.) have become common benign materials for inhibiting the generation of AMD due to their high neutralization potential [66,67,68]. The addition of these alkaline materials occurs during neutralizing reactions. the formation of secondary minerals (sulfate, carbonate, and hydroxide) can fix the dissolved metals through adsorption and co-precipitation [69,70]. A column experiment was filled with the mixture of pyrite and fly ash. The kinetic study confirmed that the pyrite oxidation rate was zero under alkaline pH when Fe(III) coating formed on the mineral surface [70]. Olds et al. [71] mixed granite powder and cement kiln ash at a volume of 4:1 as the overlay, effectively limiting the diffusion of oxygen with a permeability of only 10^−7^~10^−6^ m·s^−1^. Cement kiln ash had an alkalinity of about 650 kg CaCO_3_·t^−1^, which was dissolved and permeated slowly by rainfall to inhibit acid accumulation of the lower tailings [72]. The mixture of waste rock with pulp and alkaline by-products from steel mills caused trace element concentrations in the leaching solution to be less than 100 μg·L^−1^ and the pH to be close to neutral (pH~2) [66].

However, the continuous consumption of alkalinity limits the long-term performance of these kinds of materials. Industrial by-products usually contain more pollution impurities, which easily cause the release of toxic substances and increase the environmental burden. The release of heavy metals and the generation of neutralized sludge are environmental threats that need to be paid more attention to. Abreu et al. [73] found that, although red mud as a covering material has sufficient alkalinity for AMD generation inhibition, heavy metals accumulated at the red mud–tailing interface, causing secondary pollution along with the permeation and migration of leachate. Generally, the applications of industrial by-products have achieved remarkable results in the short-term validation, but the secondary pollution problems are inevitable, especially for the areas that need to be reclaimed after remediation, and the migration and enrichment of heavy metals should be strongly considered.

### 2.3. Organic Reactive Barriers (ORB)

**Figure 7:**
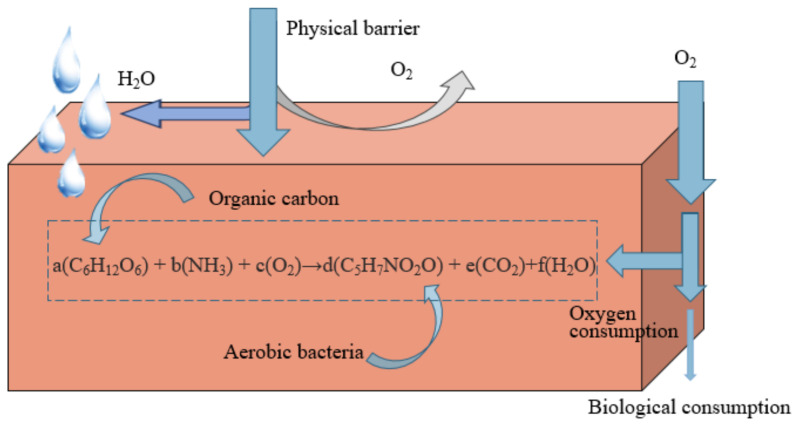
Schematic diagram of oxygen consumption coverage considering microbial growth.

The use of ORB to prevent oxygen penetration into the underlying tailings pond is an advanced technology in covering schemes as of recently. On the one hand, the physical advantages of the material itself ensure the physical barrier of external water and oxygen [74]. On the other hand, the metabolic reactions by internal microorganisms consume diffusive oxygen [9]. These two reasons cause oxygen depletion in the contact gap between the underlying sulfide minerals (Figure 7). Compared with other cover materials, organic cover materials have the advantages of low permeability, high cation exchange capacity and high alkalinity, thus limiting sulfide mineral oxidation and AMD generation [9]. Organic materials such as sawdust [75], straw, paper pulp and municipal waste compost [76,77] were used as organic cover layers, playing significant roles as oxygen barrier and acid-base modulator. However, the degradation of organic matter is rapid, and the long-term effectiveness of the covering needs a frequent supply of organic carbon-rich materials. Nason et al. [78] comfirmed that 20% of biological sludge was consumed within two years. At present, although the ORB cover has prominent oxygen barrier effects than single-layer cover in theory, there are more constraints in practice, such as the influence of temperature and pH on biological activity [79], the reduction of degradable substances on the treatment efficiency, and the impact of mine environment on microbial proliferation and variation.

Furthermore, organic reactive materials can be used as a benign soil environment regulator. The decomposition process of organic covering can provide necessary nutrients (N, P, K, Ca, Mg, etc.) for plant growth, increase soil organic matter, and provide fertility for the soil environment. It can also create a suitable environment in the covering area to promote the proliferation of microorganisms. However, the use of organic rich waste can lead to risks. For example, organic cover may induce the reductive dissolution of secondary minerals, such as iron hydroxide, resulting in the release of toxic elements (As, Cd, Cu, Pb and Se). It may also promote the activity and proliferation of pro-oxidation-bacteria, resulting in adverse effects opposite to the oxygen barrier, and more attention should be paid to the environmental risks, especially for the use of sewage sludge. For example, NH^4+^ in sludge will lead to the formation of nitrate [79], leading to the oxidation of pyrite and promoting the generation of AMD. In addition, pathogenic microorganisms also have the risk of migration along with the surface runoff.

## 3. Future Perspectives

With the progress of industrial development, increasing attention has been paid to the management of mine environment, and increasingly research has been performed with tailings covering technology. However, further development is needed from laboratory research to engineering application, and the main directions for further research are as follows:

(1) Most of the tailings covering schemes are limited to short-term research and lack of long-term problem monitoring. Therefore, it is necessary to conduct relatively long-term simulation experiments in the future research, and then it is necessary to predict the long-term treatment performance with appropriate geological simulation software so as to verify the lasting effectiveness of the scheme.
(2) The availability of water and oxygen is greatly reduced after physical covering treatment. However, the internal reactions caused by covering, such as side reactions, the proliferation and activity changes of aerobic and anaerobic bacteria, and the protective effect of precipitation coating on the surface of particles, magnify the microscopic oxidation process of sulfide minerals. Thus, the micro-oxidation process after the implementation of the technology should be studied, and the periodic oxidation reactions of sulfide minerals under different conditions and different oxidation states are encouraged to be analyzed.
(3) The management of mine environment should focus on sustainability, in which ecological restoration is an important part. Therefore, the tailing covering system can be combined with vegetation restoration as synergistic technology for both pollution control and ecological restoration.
(4) The introduction of cheap materials into the treatment scheme has showed great economic advantages. The long-term stability, as well as the leaching and migration of harmful substances and harmful side reactions of new materials, especially industrial wastes, can be further investigated in depth. In addition, the application of new materials and new technologies can be comprehensively evaluated for their environmental impacts.

## 4. Conclusions

The oxidation of sulfide minerals will lead to the continuous production of AMD, and the pollution is serious and lasting. The most effective way to suppress this pollution is to remove one of the basic reactants (oxygen, water, iron, microorganisms, etc.) required for the oxidation reaction. From the technical consideration of source control, tailing cover technology is preferred. Wet cover, dry cover, and organic reactive barriers are the basic types of tailing cover schemes. Wet covers are suitable in areas with natural water lakes, dams, and low water evaporation because water is required as an oxygen barrier. The application of the dry covers scheme has strict requirements on materials, and the covering materials used require strong stability and little secondary pollution. Organic reactive barriers are an innovative method that combines physical barriers with microbial oxygen consumption metabolism. The double barrier of cover minimizes the oxidation efficiency of sulfide, but the degradation consumption of biosolids limits the sustainability of organic reactive barriers. Therefore, organic cover is suitable for a good environment in the covering area and promotes the proliferation of a large number of microorganisms. In addition, the organic covering material must have continuous alkaline penetration and microbial metabolism. In this review, we have aimed to provide an overview about different tailings covering schemes and suggest direction for future work. It can help in guiding future studies on the development of new covering materials and technologies to controlling pollution from AMD.

## Data Availability

The data involved in this review are from published literature.
